# Adult T cell leukaemia/lymphoma (ATL) in pregnancy: A UK case series

**DOI:** 10.1002/jha2.142

**Published:** 2020-11-25

**Authors:** Leila Motedayen Aval, Mary Boullier, Hermione Lyall, Graham P. Collins, Robert Ayto, Dominic F. Kelly, Richard S. Tedder, Simon B. Drysdale, Graham P. Taylor, Lucy B. Cook

**Affiliations:** ^1^ Section of Virology, Imperial College London UK; ^2^ Department of Paediatrics St George's University Hospitals NHS Foundation Trust London UK; ^3^ Department of Paediatric Infectious Diseases Imperial College Healthcare NHS Trust London UK; ^4^ Department of Haematology Oxford University Hospitals NHS Foundation Trust Oxford UK; ^5^ Department of Haematology Portsmouth Hospitals NHS Trust Hampshire UK; ^6^ Oxford Vaccine Group Department of Paediatrics University of Oxford Oxford UK; ^7^ NIHR Oxford Biomedical Research Centre Oxford UK; ^8^ Centre for Haematology Imperial College Healthcare NHS Trust London UK; ^9^ National Centre for Human Retrovirology Imperial College Healthcare NHS Trust London UK

**Keywords:** antiretrovirals, ATL, HTLV‐1, pregnancy, screening

## Abstract

**Introduction:**

Chronic infection with human T‐cell lymphotropic virus type‐1 (HTLV‐1) may result in aggressive adult T‐cell leukaemia/lymphoma (ATL) in 4‐6% carriers. The majority of this risk arises in carriers infected during infancy, and so each infant has ∼25% lifetime risk. Other risk factors include a family history of ATL. Antenatal HTLV‐1 screening is not undertaken in the UK.

**Methods:**

Here we describe four cases of ATL diagnosed during pregnancy and describe strategies to minimise HTLV‐1 transmission to neonates.

**Results/conclusion:**

These cases highlight undiagnosed HTLV‐1 in pregnancy which allows ongoing mother to child vertical transmission and risk of future ATL. We recommend the UK National Screening Committee incorporate HTLV‐1 serology into antenatal screening.

## 1 INTRODUCTION

Human T‐cell lymphotropic virus type‐1 (HTLV‐1) is a potent carcinogenic human delta retrovirus that causes aggressive adult T‐cell leukaemia/lymphoma (ATL) in 2.5‐5% of infected persons [1]. Neonatal acquisition of HTLV‐1, mainly accounted for by breastfeeding, is closely associated with ATL in later life, and thus the 4‐6% lifetime risk of developing ATL occurs principally in the 20% of infections which arise from mother to child transmission [2]. Thus, each infected infant has ∼25% life time risk of developing ATL, making this one of the most important routes to be addressed for preventing ATL [3]. In spite of this, world‐wide only Japan and French Guiana currently implement HTLV‐1 antenatal screening [4, 5]. Furthermore, HTLV infection and ATL cluster in families, and so it is both timely and appropriate to eradicate HTLV‐1 transmission within affected families [1].

Since the 1987 implementation of antenatal screening in the Nagasaki region of Japan, HTLV‐1 seroprevalence in mothers has reduced from ∼5% to 1% and vertical transmission from 20.3% to 2.5% [6]. This success is attributed to the avoidance of breastfeeding by infected mothers, counselling for people living with the infection and the provision of training for healthcare workers [6]. In 2017, the UK National Screening Committee rejected an antenatal screening programme in part due to lack of evidence that current screening assays are effective in pregnancy but it has since been demonstrated that pregnancy does not adversely impact highly specific and sensitive HTLV diagnostic tests and are cost effective in the UK [3, 7].

ATL in pregnancy has been reported five times as individual case reports since first described in 1977, two cases from USA and three from Japan [8, 9, 10, 11, 12]. In Japan, the median age of ATL diagnosis is 67.5 years [5], and so presentation of ATL in pregnancy is expected to be a relatively rare event. By contrast median age of presentation of ATL in South America, USA and Caribbean is significantly lower with median age of 40‐50 years and in Brazil, ∼20% cases present in those less than 40 years, suggesting that ATL in pregnancy is relatively more common in HTLV‐1 endemic nations other than Japan [13, 14]. The previous described reports are of three cases of acute ATL and one lymphoma case all managed with chemotherapy, and the mothers typically died soon after delivery. Fuchi et al described a chronic unfavourable case in a pregnant woman, who was previously known to be an asymptomatic carrier (diagnosed in a previous pregnancy), and the pregnancy was terminated at 15 weeks which was reported to be associated with favourable clinical outcome [9]. ATL is a heterogeneous disease, and a recent consensus report suggested patients with lymphoma subtypes should be managed with chemotherapy‐based protocols [15, 16]. However, leukemic disease (acute or chronic), may be managed with zidovudine and interferon‐alpha (ZDV/IFN‐α), where available [16]. ZDV/IFN‐α is not approved for treatment of ATL in Japan, and so its use in pregnancy to manage ATL has not been previously reported.

Here we describe four cases of ATL presenting during pregnancy in the UK (one acute, one lymphoma, and two chronic unfavourable) including cases managed with ZDV/IFN‐α, with a focus on the strategies used to treat the mothers to reduce vertical HTLV‐1 transmission, and highlight an undiagnosed HTLV‐1 population in a non‐endemic country. Cases are summarised in Table 1.

**TABLE 1:** Clinical summary

Case No.	Year	Patient age (years)	Patient ethnicity	ATL type	Known to be HTLV‐1 infected prior to ATL diagnosis?	ATL treatment	Maternal Anteretrovirals received?	Mode of delivery	Weeks of gestation at birth	Maternal outcome	Infant outcome	Infant breastfed	Infant HTLV‐1 infected	Infant received ARV prophylaxis
1	2006	40	Caribbean	Acute	No	CHOP (post delivery)	Yes (ZDV)	C/S	29	Died shortly after delivery	Survived	No	DNA PCR ‐ve out to 7 months. Sero +ve and DNA PCR +ve from 15 months	ZDV 6 weeks
2	2016	38	African	Chronic ‘unfavourable’	No	ZDV/IFN‐α	Yes (ZDV + raltegravir)	C/S	39	Acute ATL 6 months post‐delivery and died	Survived	No	HTLV DNA not detected at 6 months	ZDV 6 weeks
3	2016	36	Caribbean	Lymphoma	No	ESHAP	Yes (ZDV)	C/S	29	Died 2 months post‐delivery	Survived	No	HTLV DNA not detected 0‐6 weeks	ZDV 6 weeks
4	2019	35	South Asian	Chronic ‘favourable’	No	ZDV/IFN‐α . Mogamulizumab 8‐weeks post‐delivery	Yes (ZDV + raltegravir)	C/S	38	Survived	Survived	No	HTLV DNA not detected at 0,6 weeks and 3 months. Still under follow‐up. Infant thriving	ZDV 6 weeks

Abbreviations: ARV, antiretroviral; CHOP, cyclophosphamide, vincristine, doxorubicin, prednisolone; C/S, caesarean section; ESHAP, etoposide, methylprednisolone, cytarabine, cisplatin ZDV zidovudine; IFN‐α, interferon‐α; +ve, positive; ‐ve negative.

### 1.1 Case 1

A 40‐year‐old pregnant female was diagnosed in 2006 with acute ATL at 28 weeks’ gestation. Her full blood count (FBC) showed: white cell count (WCC) 76.4 × 10^9^/L (normal range: 5.6‐16.9 × 10^9^/L), lactate dehydrogenase (LDH) 1382 IU/L (normal range: 125–243 IU/L), normal haemoglobin and platelets. Her blood film showed >5% abnormal pleomorphic lymphocytes with typical flower cells, and flow cytometry revealed a clonal CD3+CD4+CD25+CD7− population with positive HTLV serology. She was commenced on steroids to control her WCC and to mature foetal lungs, and with oral ZDV as part of ATL treatment and to reduce vertical transmission. She underwent emergency caesarean section 9 days later for maternal indications (WCC 172 × 10^9^/L). The maternal HTLV‐1 proviral load (PVL) (proportion of infected mononuclear cells per 100 cells) was unknown, and the mother died shortly after delivery due to sepsis during first CHOP (cyclophosphamide, doxorubicin, vincristine, and prednisolone) chemotherapy. The infant was formula‐fed and treated with ZDV post‐exposure prophylaxis for 6 weeks (the first 2 weeks were administered intravenously). At birth and age 7 months, HTLV‐1 PVL was not detected but was detected (PVL 0.01%) at 15 months, and she became HTLV‐1 seropositive. The child is now aged 13 years old and is clinically well with a stable low PVL 0.01‐0.03%.

### 1.2 Case 2

A 38‐year‐old asymptomatic woman was diagnosed in 2016 with chronic ‘unfavourable’ ATL at 7 weeks’ gestation with FBC: WCC 16.8 × 10^9^/L (normal range: 5.6‐16.9 × 10^9^/L), normal haemoglobin and platelets, LDH 516 IU/L (normal range: 125‐243 IU/L) and normal calcium. Film morphology showed occasional flower cells and frequent atypical lymphocytes. Flow cytometry and T‐cell receptor gene rearrangement studies revealed a clonal CD3+CD4+CD25+CD7‐ population with positive HTLV serology and HTLV‐1 PVL 94%. She was commenced on ZDV 250 mg twice daily and subcutaneous interferon‐α (IFN‐α) three million units three times per week with successful normalization of the lymphocyte count within weeks. From 24 weeks gestation onwards maternal HTLV PVL was stable low load between 6‐9%. Raltegravir (integrase inhibitor) 400 mg twice daily was added to ZDV/IFN‐α at 34 weeks’ gestation to further reduce the risk of vertical transmission. There were no pregnancy complications, and the infant was born by planned pre‐labour caesarean section at 39 weeks’ gestation. The infant was formula‐fed and received 6 weeks oral ZDV. HTLV‐1 deoxyribonucleic acid (DNA) was not detected at day 1, 6 weeks or 6 months. Six months post‐delivery, the mother's ATL accelerated to an acute ATL subtype and was refractory to both ZDV/IFN‐α and salvage ESHAP (etoposide, methyl prednisolone, cisplatin and cytarabine) chemotherapy and died. The baby returned to family overseas and is lost to follow up.

### 1.3 Case 3

A 34‐year‐old female was diagnosed with ATL lymphoma subtype overseas and treated there with chemotherapy (eight cycles of CHOP). Two years later in 2016, she presented in the UK with a gastric bleed, and a gastric biopsy revealed relapsed ATL lymphoma diagnosed at 24 weeks’ gestation. She was treated with two cycles of ESHAP chemotherapy, with concurrent ZDV 250 mg twice a day and subcutaneous IFN‐α 3 million units three times per week. A caesarean section was performed at 29 weeks’ gestation for foetal growth cessation (PVL 1.2% at time of delivery). The infant was severely unwell after birth with renal failure, cardiac failure and pancytopenia, attributed to maternal chemotherapy. The infant was formula‐fed and received 6 weeks of oral ZDV. HTLV‐1 DNA was not detected at day 1 or week 6. The mother died from refractory ATL 2 months post‐delivery, and the baby returned to family overseas and is lost to follow up.

### 1.4 Case 4

A 35‐year‐old female was diagnosed with chronic ‘favourable’ ATL at 12 weeks’ gestation following a routine blood count. Her FBC revealed: WCC 17.2 × 10^9^/L (normal range: 5.6‐16.9 × 10^9^/L), normal haemoglobin and platelets, corrected calcium and LDH. Her blood film showed >5% abnormal pleomorphic lymphocytes with positive HTLV‐1 serology. Diagnostic Immunophenotyping showed CD3+CD4+CD25‐CD7‐ population and HTLV‐1 PVL 45%. She was commenced on ZDV 250 mg twice daily and subcutaneous pegylated IFN‐α‐2b pegasys 1.5 mcg/kg weekly at the start of the second trimester. Raltegravir 400 mg twice daily was added at 32 weeks. There was not a significant response to therapy despite escalating doses of ZDV to 500 mg twice daily and IFN‐α 6mcg/kg, and so an elective caesarean was expedited at 38 weeks’ gestation. The infant was formula‐fed and completed 6 weeks oral ZDV syrup. At delivery neonatal HTLV PVL was undetectable and remained undetectable at 6 weeks and 3 months. The baby remains well and is under active follow‐up with repeat testing planned at 18 months. Following delivery, the mother was treated with mogamulizumab 1 mg/kg due to lack of response to ZDV/IFN‐ α. Following one dose, haematological remission was achieved, and PVL was reduced to 0.06%. She has received a total of three doses, currently stopped due to mild skin rash and remains in a persistent haematological remission 8 months later.

## 2 DISCUSSION/CONCLUSION

Two of these patients were diagnosed with chronic leukaemic ATL in the first trimester of pregnancy following the incidental finding of lymphocytosis (cases 2 and 4). Both mothers were treated with ZDV/IFN‐α which are known to be relatively safe in pregnancy, the aim being to induce remission and reduce the risk of vertical HTLV‐1 transmission. In both cases raltegravir, which inhibits HTLV‐1 integrase in vitro and readily crosses the placenta, was added in the 3rd trimester to protect foetal mononuclear cells exposed to maternal cells during the perinatal period [17]. Both infants were delivered at term by pre‐labour caesarean section, exclusively formula‐fed and treated for 6 weeks with ZDV (as per human immunodeficiency virus (HIV) practice) [18]. Both infants were thriving, and their blood remained HTLV DNA polymerase chain reaction (PCR) negative at last follow‐up, although one infant is lost to longer term follow up. By contrast, two mothers presented in late 2nd and early 3rd trimester with aggressive ATL, both refractory to chemotherapy and infants requiring emergency delivery. In case 1, where the mother had high WCC and presumably high HTLV PVL, the infant was found to be anti‐HTLV‐1 antibody positive at 15 months. In case 3, the baby remained blood‐HTLV‐1 DNA negative at last follow‐up, although long‐term outcome is unknown.

The diagnoses of both cases of chronic ATL early in pregnancy, whilst asymptomatic, allowed time to undertake strategies to reduce transmission and reflects the ‘tip of the HTLV‐1 iceberg’ since unscreened pregnant women in the UK with asymptomatic HTLV‐1 infection are unknowingly transmitting infection, chiefly through breastfeeding which is promoted by healthcare bodies in the UK. It is notable and reassuring that breastmilk banks do screen their donors for HTLV‐1.

In the UK, HIV, hepatitis B and syphilis are the only infectious diseases routinely screened for in pregnancy, and the exclusion of HTLV‐1 from this list is difficult to justify given its associated fatal diseases. Additionally, a recent meta‐analysis revealed that asymptomatic HTLV‐1 infection results in 57% increased risk of premature death that is independent of ATL and HTLV‐1 associated myelopathy, the two diseases commonly associated with HTLV‐1 [19].

HTLV‐1 remains neglected by health agencies globally, has not been recognised as an oncogenic virus by the National Cancer Institute and only very recently acknowledged by the WHO as a sexually transmitted infection [20]. With all the evidence supporting the benefit of antenatal screening programmes, we urge the national screening committee to reconsider their decision which excludes HTLV‐1 in the UK antenatal screening programme. A policy which inevitably fails to offer the HTLV‐1 infected mother, her siblings and family members, and her future children appropriate knowledge and clinical interventions to mitigate its preventable serious disease burden.
